# Dynamic Testing in a Heterogeneous Clinical Sample: A Feasibility Study †

**DOI:** 10.3390/bs15101342

**Published:** 2025-09-29

**Authors:** Ynès Hendriks, Bart Vogelaar, Roos van Heeswijk, Jochanan Veerbeek, Wilma Resing, Loes van Aken, Jos Egger

**Affiliations:** 1 Centre of Excellence for Neuropsychiatry, Vincent van Gogh Institute for Psychiatrie, 5803 AC Venray, The Netherlands; 2 Donders Institute for Brain, Cognition and Behaviour, Radboud University, 6525 XZ Nijmegen, The Netherlands; 3 STEVIG Specialized and Forensic Care of People with Intellectual Disabilities, Dichterbij, 5807 EA Oostrum, The Netherlands; 4 Department of Developmental and Educational Psychology, Leiden University, 2311 EZ Leiden, The Netherlands; 5 Education and Child Studies, Leiden University, 2311 EZ Leiden, The Netherlands

**Keywords:** dynamic testing, neuropsychological assessment, learning, contextual neuropsychology, psychiatric disorder

## Abstract

This study evaluated the feasibility of including a computerized dynamic test of analogical reasoning in standard neuropsychological assessments in a heterogeneous psychiatric population. The participants were 40 adult patients (*M*_age_ = 33.15 ± 12.27, range 19–68; 60% male) enrolled in specialized mental health and forensic care programs in The Netherlands, who were randomly assigned into either a training, a passive, or a control group. A pretest–training–posttest paradigm was used for the training group, and the dynamic test consisted of 26 items of the A:B::C:? type. In terms of practical use, it was found that the administration time varied largely, and 22% of the data was lost due to drop out or technical malfunctions. Test–retest reliability was acceptable for the training group (*r* = 0.61) and good for the practice and control groups (resp. *r* = 0.88 and 0.80). A statistical trend was observed for the training vs. practice group (*Z* = −1.598, *p* = 0.055), but not for the training vs. control group (*Z* = −0.839, *p* = 0.201). It was concluded that an indication of training effectiveness was found; however, in this clinical sample, the applicability of the current form of the dynamic test is still limited. Several modification options are discussed.

## 1. Introduction

In the field of clinical neuropsychology, a wide array of instruments are used to capture cognitive processes such as attentional control, perceptual processing, learning and memory, abstract thinking, and executive functioning (Vakil, 2012). Such assessments provide insight into someone’s cognitive functioning and can guide diagnosis, psychoeducation, treatment selection, and evaluation alongside designs of rehabilitation programs (Kessels, 2019; Lezak et al., 2012; Vakil, 2012). Although using these tests has certain advantages, such as the ease of administration and interpretation and the ability to compare performance against standard scores, how ecologically valid the results of the clinical neuropsychological assessment are—or, in other words, how representative these assessments are for performance in everyday life and how much predictive value they have for future behavior (Lezak et al., 2012; Sbordone, 2008; Wilson, 1993)—is questioned. Most of these tests are of a static nature. These tests often have a one-session format, with individuals solving cognitive tasks after a short (standardized) instruction. Often, provision of additional help or feedback is not permitted, thus providing a snapshot of what an individual has already acquired in terms of knowledge and skills. As such, static tests are said to provide limited information about the cognitive processes underlying test performance, since test performance depends on an interconnected system of cognitive processes (Tosi et al., 2020). An accurate interpretation of test results is feasible only if behavioral changes during the test are taken into account. These are primarily observed by clinicians and are inherently subjective in nature (Oomens et al., 2023). Consequently, static testing is mainly assessing knowledge that has or has not been acquired in the past and essentially does not include information about the learning potential of the individual: the capacity to learn in the present or future (Resing, 2016).

The concept of learning potential implies a distinction between the cognitive expertise that has been developed so far and the latent cognitive expertise—cognitive skills that can be developed in the (near) future. The latter resembles what Vygotsky (Vygotsky, 1978) described as the Zone of Proximal Development (ZPD)—the difference between what a person can do independently (actual level of development) and what a person can do with a more knowledgeable/skilled other person (potential level of development). By making use of static testing only, the level of potential development cannot be assessed adequately, which can differ from the actual level of development due to, for instance, social-cultural influences such as educational opportunities. For instance, in children with developmental delays, especially when paralleled with behavioral problems and psychopathology, it was found that scores on intelligence tests only partially reflected learning potential (Carlson & Wiedl, 2000; Resing et al., 2012). The levels of potential and actual development should therefore be measured in different ways, reflecting why dynamic tests were designed to complement static tests in assessing cognitive functioning. Dynamic testing is an assessment approach that evaluates an individual’s learning potential by examining their ability to learn and apply new skills or knowledge when provided with feedback, guidance, or instructions during the testing process. This method emphasizes the process of learning rather than static performance levels, offering insights into cognitive flexibility and adaptability (Sternberg & Grigorenko, 2002).

Since dynamic tests provide information about the potential for future learning (Boosman et al., 2016), it may be useful to include dynamic testing principles in clinical neuropsychological assessment procedures. To measure learning potential, some dynamic tests use inductive reasoning tasks—and, in particular, analogical reasoning—since it is important in learning and reasoning in general. Analogical reasoning refers to the ability to find similarities according to relations that hold across different sets of elements (Gentner & Maravilla, 2017). Dynamic testing of analogical reasoning abilities has proven to be of added value in predicting (future) school performance in children (Resing, 1993; Stevenson et al., 2014). In these tests, a pretest–training–posttest paradigm can be used, in which a test is administered before and after a training. During this training, feedback can be given according to the graduated prompting principles, which concerns provision of hints or prompts in a gradual sequence of increasing explicitness, starting with general metacognitive prompts that become increasingly more task-specific and cognitive, in order to learn the rules needed to solve the problem correctly (Brown & French, 1979). Dynamic testing therefore enables exploring individual differences in (1) instructional needs, i.e., the amount and type of help an individual needs during training, (2) learning potential, here the ability to benefit from feedback, and (3) transfer potential, i.e., the ability to generalize newly learned skills. In children, it has already been demonstrated that graduated prompting leads to enhanced learning compared to repeated practice only (Resing et al., 2019; Touw et al., 2020).

The advantages of dynamic testing principles in neuropsychological assessment procedures have already been demonstrated in typically aging elderly people and in several clinical populations, mainly including patients with a schizophrenia spectrum disorder, elderly patients with mild cognitive impairment (MCI) or neurodegenerative disorders, and patients with brain injuries of various causes. In summary, these studies compared dynamic measures to static scores, which indicated better discrimination between typically and non-typically aging elderly people (Fernández-Ballesteros et al., 2003; Küster et al., 2016; Uttner et al., 2010; Wiedl et al., 2001) and superior predictive validity with regard to rehabilitation potential in patients with a schizophrenia spectrum disorder—in favor of the dynamic test scores (Davidson et al., 2016; Sergi et al., 2005; Wiedl et al., 2001). Additionally, including a dynamic test in neuropsychological assessments proved valuable for evaluating learning potential of both patients with traumatic and non-traumatic acquired brain injury. In both groups, patients who received training between the pre- and posttest showed greater improvement on the posttest than controls who did not (Boosman et al., 2014; Toglia & Cermak, 2009).

As yet, research on dynamic testing mainly focuses on children and adolescents (within the field of school psychology) or on specific psychiatric populations. In general, neuropsychological assessment procedures are widely applied in clinical psychiatric practice. Information about the added value of dynamic testing in neuropsychological assessment in clinical practice is lacking, even though it is considered to be of added value for optimization and personalization of, for example, psychotherapy. The present study will be exploratory in nature, focusing on the feasibility of including a dynamic test in standard neuropsychological assessments in a heterogeneous psychiatric population. A shortened version of a computerized. standardized dynamic test of analogical reasoning (Hosenfeld et al., 1997; Vogelaar et al., 2019, 2021) will be used. The feasibility of this test has already been demonstrated in non-gifted and gifted children, as trained children improved more than children in the control group. However, it has not yet been used in an adult heterogeneous, psychiatric population. Therefore, the current study will evaluate the applicability of the dynamic test in this population. In doing so, psychometric properties and initial experience such as randomization, drop out, and practical use of the dynamic test in this clinical population will be evaluated. Moreover, the usability of a dynamic test in this population will be further explored by focusing on potential training effects and the relationship between the dynamic test and a number of static measures, including measures of intelligence, memory, and learning.

## 2. Materials and Methods

### 2.1. Participants

All participants in this study were patients of the specialist mental health care programs of the Vincent van Gogh Institute for Psychiatry in Venray or of STEVIG, Specialized and Forensic Care in Oostrum, the Netherlands, for whom clinical neuropsychological assessment was indicated. Receiving health care in either of these facilities requires referral by a general practitioner or a mental health specialist, such as a psychologist or psychiatrist. Referral is possible for people who suffer from complex psychiatric problems combined with cognitive complaints. The original sample consisted of 51 adult participants. Exclusion criteria were an age below 18 years, an (estimated) Full-Scale Intelligence Quotient (FSIQ) lower than 70, and/or not mastering the Dutch language. Ultimately, 40 patients were included (*M*_age_ = 33.15 ± 12.27, range 19–68; 60% male). Data of the other eleven participants were excluded because they were not stored properly (*n* = 9) or because participants dropped out early (*n* = 2). Participants were randomly and manually assigned to one of three groups: (1) a training group, who received training between the pre- and posttest, which consisted of six guided items (i.e., help was provided when patients did not solve the items independently) (*n* = 14); (2) a practice group, who completed the same six items between the pre- and posttest as the training group but did not receive any guidance (*n* = 15); and (3) a control group, which only completed the pre- and posttest (*n* = 11). Further details on the different phases of the dynamic test can be found in Table 1 and in Section 2.3.

### 2.2. Procedures

During or after the clinical neuropsychological assessment, participants were informed of the content and objectives of the study by means of an information folder. Participation was voluntary, and no compensation was offered. When the participants indicated that they were willing to participate in the study, they were asked to sign an informed consent form. Information regarding age, gender, level of education, and relevant institution and department was collected by the assessor and noted on a form. The level of education of the participants was classified according to the classification system of Verhage (Bouma et al., 2012). Information on medication use and psychiatric and medical history was collected for future reference. After randomization, the dynamic test was introduced to the participant, who independently completed the computerized test in the presence of an assessor. In all cases, administration was performed by a trained psychologist or postgraduate student in psychology. All information was processed anonymously in a database. The data presented in this study are available on request from the corresponding author due to privacy reasons. Approval for the study was given by the Vincent van Gogh Institutional Review Board (EM/hl/2019.00.03 and 19.01817). No potential risks were identified.

### 2.3. Materials

Learning potential was measured using a dynamic version of an analogical reasoning test. This test was computerized and standardized and has previously been used in studies of Vogelaar and colleagues (Vogelaar et al., 2019, 2021); items were selected out of a test battery that was originally created by Hosenfeld, Van den Boom, and Resing (Hosenfeld et al., 1997). After a short initial pilot, it was concluded that the dynamic test was too strenuous for the participants in this clinical sample. Therefore, in the current study, a less cognitively demanding, shortened version was used, reducing the number of test items from 52 to 20 (for the control group) or 26 (for the training and practice group) in total. Internal consistency reliabilities for the pre- and posttest of the original 52-item test were high (resp. α = 0.93 and α = 0.95 to 0.96; Vogelaar et al., 2019). The potential impact of shortening the test to 20/26 items on validity and reliability will be examined in the current study.

**Figure 1:**
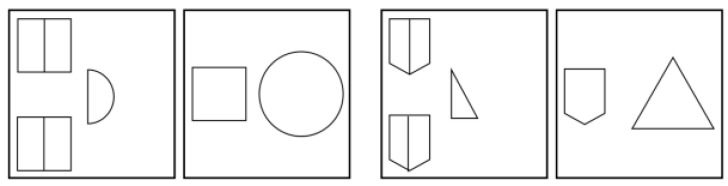
Example of an analogical reasoning test item with correct answer (last frame).

The analogical reasoning test consisted of items of the A:B::C:? type. Each item of the dynamic test consisted of two pairs of frames. The first two frames were filled with geometric shapes and referenced how these shapes must be transformed to give the correct answer. The fourth frame had to be constructed by the participant by transforming the shapes in the third frame according to the ‘rules’ shown in the first two frames. For an example, see Figure 1. The participants had to choose the correct geometrical shape (triangle, hexagon, circle, square, ellipse, or pentagon) and then had to use several buttons for rotating, mirroring, bisecting, and changing the size of the shapes. Buttons for undoing one step at a time or all previous steps at once were also available.

All participants completed a pre- and posttest, which consisted of 10 items, increasing in difficulty from item 1 to 10. A total score was calculated for both the pre- and posttest based on the total number of correctly answered items per phase. The participants that were assigned to the training and practice group completed six items between the pre- and posttest either guided or unguided, respectively. These training and practice items consisted of new analogy items that were comparable to those administered in either the pre- or posttest. Instructions were provided by the same pre-recorded, female voice for all testing conditions. During the training, feedback was automatically provided according to the graduated prompting principles, meaning that the participants were offered hints gradually, with increasing explicitness. If the participant gave the correct answer at the first attempt, no feedback was provided, and the next item was presented immediately. However, when the participant did not give the correct answer, the first hint was given, and so on, until a maximum of four different hints was reached, ranging from a metacognitive prompt, two cognitive prompts, to step-by-step modeling instructions. After the last hint was given, the participant had one last chance to give the correct answer. The program was coded to automatically score responses. Logged variables included whether an item was answered correctly or incorrectly and the administration time per item. During the training phase, the number of hints required by the participant was also recorded. The test was programmed on a laptop as a stand-alone application, with data subsequently transformed and stored on a USB drive.

Static measures that are part of the standard clinical neuropsychological assessment procedures within the two institutes were selected based on their psychometric properties. First, intelligence was measured with the fourth edition of the Dutch version of the Wechsler Adult Intelligence Scale (WAIS-IV-NL; Wechsler, 2012a, 2012b). In addition to FSIQ scores, index scores for Verbal Comprehension (VCI), Perceptual Reasoning (PRI), Working Memory (WMI), and Processing Speed (PSI) were calculated. Other static measures included the Dutch version of the Rey Auditory Verbal Learning Test (RAVLT; Mulder, 2012), used to measure verbal-auditory learning and memory, and the Location Learning Test (LLT; Kessels et al., 2014), used to measure visuospatial learning and memory. For an overview of the variables that were included for these static measures, see Table 2.

### 2.4. Statistical Analyses

To examine whether groups differed in age, FSIQ, and pretest scores, a multivariate analysis of variance (MANOVA) was used. To identify potential differences in gender, level of education, and relevant institutions/departments, three χ^2^ tests were conducted. Moreover, to explore the psychometric properties of the dynamic test, internal consistency reliabilities were calculated for the pre- and posttest. Additionally, test–retest reliability was calculated for all three groups (training, practice, and control) and transformed to z-scores according to Fisher to determine whether test–retest reliability differed significantly. To explore whether a training effect had taken place, a repeated measures analysis of variance (RM ANOVA) was performed. Hereby, the accuracy scores on the dynamic test was used as a dependent variable, time (pretest and posttest) was used as a within-subject factor, and group (training, practice, and control) was used as a between-subject factor. In addition, to look into the potential coherence between dynamic test scores on the one hand and static test scores on the other hand, bivariate correlation analyses were executed.

## 3. Results

### 3.1. Feasibility

The original sample consisted of 51 participants. The final sample consisted of 40. Two participants decided to stop during the dynamic test assessment. They perceived the test as too difficult, too long, frustrating, or a combination of these. Although not all other participants provided feedback, their behavior appeared to align with these reported experiences. For instance, some drew on the test form (such as a face with eyes, a nose, and mouth). Several participants had difficulties in operating the computer, like double-clicking, moving geometric shapes with a mouse, and/or remembering button functions, which sometimes led to accidentally clearing the fourth frame, requiring them to start over. Consequently, the dynamic test took up to 2.5 h in some cases, despite the aim of completing each phase of the test within 20 to 30 min. See Table 3 for the descriptives of the administration times for each phase of the dynamic test.

For nine participants, data was not properly saved due to USB malfunctions or failed screenshot captures, rendering their answers unverifiable and excluding them from ana-lysis. Additionally, six participants received excessive hints during training because the test could not reliably distinguish between geometric shapes in the fourth frame. Attempts to address these issues during data collection were unsuccessful. As a result, training phase scores did not reliably reflect instructional needs and were therefore excluded from the final analyses.

A Mann–Whitney U test was conducted to determine whether there were differences in pretest scores for the included (*n* = 40) versus excluded participants prior to conducting our analyses (*n* = 6; the data of the remaining five participants could not be included because they were unavailable). No significant differences in pretest scores were found between these two groups (*U* = 62.50, *p* = 0.059).

A multivariate analysis of variance indicated no significant differences between groups (training, practice, and control) for the dependent variables (age, FSIQ, and pretest scores) (*F*(3, 68) = 2.16, *p* = 0.057). Furthermore, χ^2^ test results indicated significant differences in gender between groups (χ^2^(2) = 6.07, *p* = 0.048) but not in level of education (χ^2^(8) = 7.64, *p* = 0.470) or involved institutions/departments (χ^2^(8) = 8.33, *p* = 0.402). These results suggest that randomization was successful.

### 3.2. Psychometric Properties

Exploration of the psychometric properties of the 26-item version of the dynamic test revealed acceptable internal consistency reliability for the pretest (α = 0.79) and good-to-excellent internal consistency reliability in all three groups for the posttest (αs ranging from 0.81 to 0.91). Additionally, test–retest reliability was acceptable for the training group (*r* = 0.61) and good for the practice and control group (resp. *r* = 0.88 and 0.80). Although pre- and posttest scores seemed to correlate more strongly in the practice and control conditions than in the training condition, differences in test–retest reliability were not significant (*Z* = −1.598, *p* = 0.055 for the training vs. practice group; *Z* = −0.839, *p* = 0.201 for the training vs. control group).

### 3.3. Learning Potential

**Figure 2:**
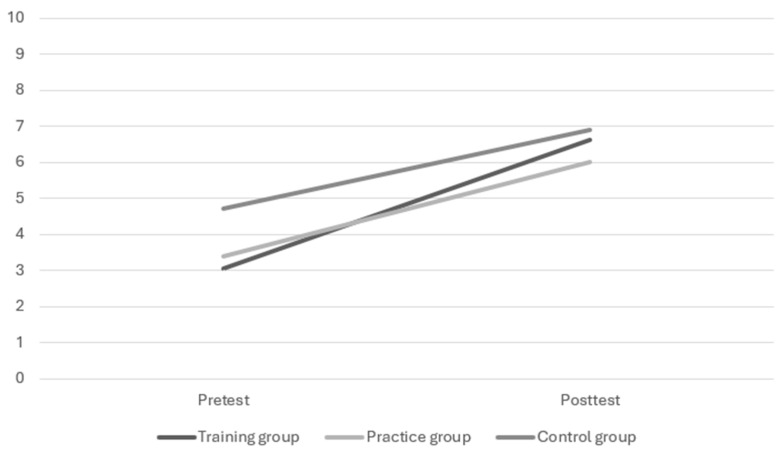
Progression paths on the dynamic test per group.

**Figure 3:**
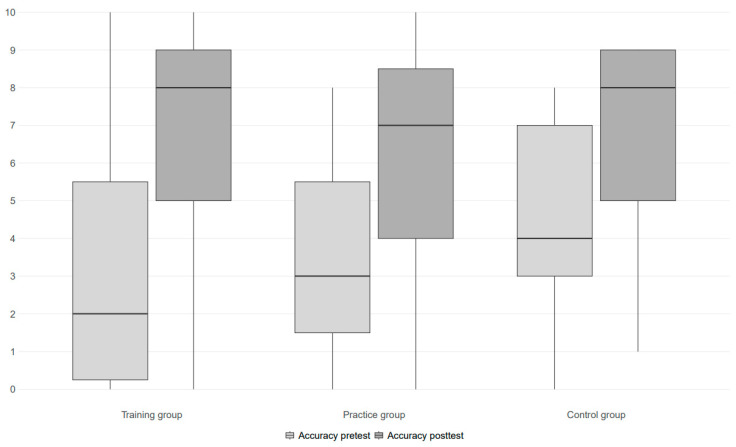
Boxplot displaying the median and variability of pretest and posttest scores across groups.

A univariate repeated measures analysis of variance showed a significant main effect of time (*F*(1, 37) = 61.05, *p* < 0.001, η_p_^2^ = 0.62). A visual check of the mean scores indicated that, in general, the mean total scores on the dynamic test were higher at posttest than at pretest for all groups (resp. *M* = 3.65 and *M* = 6.48). The time x group interaction was not significant with a medium effect size (*F*(2, 37) = 1.31, *p* = 0.281, η_p_^2^ = 0.07). Throughout the sample, there was an observed increase in the mean total scores between the pre- and posttest, but the size of this increase did not differ significantly between the training, practice, and control groups. For the progression paths on the dynamic test for each group (resp. *M* = 3.57, *M* = 2.60 and *M* = 2.18), see Figure 2. Furter visual inspection of the data is provided in Figure 3.

### 3.4. Correlations Between the Dynamic and Static Measures

For all descriptives of the dynamic and static measures, see Table 4. Bivariate correlation analyses showed significant correlations between pretest scores on the dynamic test on the one hand and all static measures on the other hand. More specifically, significant correlations were found between pretest scores and FSIQ scores (*r* = 0.715, *p* < 0.001), VCI scores (*r* = 0.557, *p* < 0.001), PRI scores (*r* = 0.675, *p* < 0.001), WMI scores (*r* = 0.565, *p* < 0.001), PCI scores (*r* = 0.507, *p* = 0.001), total number correct on the RAVLT (*r* = 0.510, *p* = 0.001), and the learning index of the LLT (*r* = 0.525, *p* = 0.001), indicating strong correlations between all variables. For the total number of errors on the LLT, a moderately strong correlation was found (*r* = −0.407, *p* = 0.012).

To explore the correlation between posttest scores on the dynamic test on the one hand and static measures on the other hand, the results were analyzed per group. In the training group, significant correlations were found between posttest scores and FSIQ scores (*r* = 0.572, *p* = 0.041), PRI scores (*r* = 0.568, *p* = 0.043), and WMI scores (*r* = 0.613, *p* = 0.026), all indicating a strong effect. Moreover, in the practice group, posttest scores correlated significantly with FSIQ scores (*r* = 0.624, *p* = 0.013), VCI scores (*r* = 0.545, *p* = 0.036), PRI scores (*r* = 0.661, *p* = 0.007), total number of errors on the LLT (*r* = −0.770, *p* = 0.001), and the learning index of the LLT (*r* = 0.710, *p* = 0.003). In addition, for the control group, significant correlations were found between posttests cores and FSIQ scores (*r* = 0.861, *p* = 0.001), VCI scores (*r* = 0.646, *p* = 0.043), PRI scores (*r* = 0.699, *p* = 0.024), and WMI scores (*r* = 0.878, *p* = 0.001). In summary, in all three groups, posttest scores correlated significantly with FSIQ scores, and this correlation was the weakest for the training group and the strongest for the control group. However, these correlations did not differ significantly (training vs. control group, *Z* = −1.392, *p* = 0.082). For an overview of all corresponding statistics, see Table 5.

## 4. Discussion

The current study investigated the feasibility of including a computerized and standardized dynamic test into standardized neuropsychological assessment in a heterogeneous psychiatric population. Beyond evaluating the psychometric properties of and initial experience with the test, it was also explored whether participants showed improvement in analogical reasoning after training and to what extent the dynamic test outcomes were related to static measures of intelligence, memory, and learning.

With regard to the psychometric properties of the test, it was found that the test–retest reliability was acceptable for the training group and good for the practice and control groups, suggesting that posttest score variability was the highest in the training group, as illustrated as well in the boxplots depicting the data for each group. Statistical analysis, however, showed no significant differences between groups in terms of improvement from pre- to posttest over time, although significance is not the main concern given the small sample size and the resulting limitations in statistical power. A qualitative interpretation of the score patterns revealed improvements from pretest to posttest in all three groups, suggesting that a testing effect cannot be entirely excluded. Nevertheless, when investigating the relationships between the static measures of intelligence and posttest scores, it was found that the relationship between FSIQ and posttest scores appeared weakest in the training group and strongest in the control group, suggesting that the inclusion of brief training may positively influence posttest performance—as we had hoped for. It should be noted, however, that FSIQ and index scores also show substantial variability and that individual differences both between and within groups deserve further in-depth investigation in future research.

Despite these promising outcomes, feasibility of including the dynamic test in standardized neuropsychological assessment is not yet self-evident. Data loss (22%) due to dropout or technical issues negatively impacted the study and would hinder clinical use as well. Instructional needs could not be addressed, as 43% of the participants in the training group required many hints. Future studies could benefit from a more in-depth investigation of participants’ instructional needs, for example by examining the relationship between instructional needs, training performance, and posttest outcomes. In addition, the test’s administration time varied largely, occasionally exceeding two and a half hours, which was too intensive for several participants. Some of them explicitly wanted to stop, while others appeared disengaged without expressing it verbally. Combined with challenges in operating the computer, these issues raise significant concerns about the test’s practicality in its current form for this heterogeneous clinical population.

The findings above are not in line with the findings of Vogelaar and colleagues (Vogelaar et al., 2019, 2021), who successfully used the 52-item version of this test in children aged 6 to 9 years. One explanation could be that using a computer might be easier for young children than older people, as from a very young age children are subjected to computerized means of testing. Also, specific cognitive difficulties, such as decreased sensorimotor skills and working memory capacity, which are often found in patients with psychiatric burden (Kushwaha et al., 2024), could make it more difficult to use a computer. However, the findings in this study showed that, at the group level, both WMI and FSIQ scores were (low) average in all three groups, although individual test scores varied widely. Nonetheless, a shorter dynamic assessment would be preferable, since the standard clinical neuropsychological assessment is already time-intensive and patients with serious mental illness are often already less resilient.

The psychometric evaluation revealed acceptable to good internal consistency reliability for the pretest and posttest, respectively. Test–retest reliability was higher for the practice and control group than for the training group, suggesting a greater degree of learning in the latter group. While the difference in test–retest reliability between the training and control group was bordering on significance, the difference between the training and practice group was not. Even though statistical analyses found no significant differences in improvement over time between groups, qualitatively interpreted gain scores suggested greater learning in the training group, with trained participants showing the most improvement from pre- to posttest. The variability within the training group should be noted, however, as it aligns with previous studies showing that dynamic test performance can also be interpreted individually (Boosman et al., 2016). These individual differences in improvement are highly relevant in clinical practice. For example, they allow individual participants to be classified as ‘learners’, who benefit from training (or another intervention), or as ‘non-learners’, who do not (Schöttke et al., 1993). This could, for instance, be beneficial in providing personalized recommendations for psychological treatment.

As might be expected, since the pretest can be considered as a static test, it was found that pretest scores on the dynamic test strongly correlate with scores of all the static measures used in this study (WAIS-IV-NL, RAVLT, and LLT). However, posttest scores were found to correlate positively with intelligence as well. Interestingly, posttest scores seemed to correlate more strongly with FSIQ scores in both control groups, when compared to the training group, indicating that posttest scores of the training group provide different information from posttest scores of the control groups. Differences in correlations between both training and control groups almost reached significance, and possible effects may be overlooked given the low statistical power resulting from the small sample size. Therefore, no conclusions can yet be drawn about the learning potential in this heterogeneous psychiatric population.

Although it can be suggested with caution that dynamic testing has potential in a heterogenous clinical sample, adjustments need to be made to the current dynamic test. As to the large variation in administration time in the current study, it might be worth investigating whether variation in administration time in a similar research sample might be related to weaknesses in participants’ cognitive skills and functions. While several studies in healthy children showed no differences in the effectiveness of a computerized or analog version of a dynamic test (Resing et al., 2011; Stevenson et al., 2011; Tzuriel & Shamir, 2002), a pencil-and-paper version of the dynamic test can be considered in the future, partly to prevent losing data due to technical failures, but more importantly to eliminate the difficulties of controlling a computer. Moreover, participants might experience more commitment to an assessor than to a computer, helping them to stay motivated. The tester–testee interaction is considered an important aspect within the ZPD, and more specifically in dynamic assessment, since it distinguishes dynamic testing from static testing. In the conventional psychometric approach, a neutral attitude of the tester is usual, whereas in dynamic assessments, an interactive setting of teaching and helping is necessary, tailored to the needs of the testee (Grigorenko & Sternberg, 1998; Schack & Guthke, 2003). In addition, making use of cut-off criteria and/or multiple-choice answers should also be considered, as this may potentially decrease the administration time of the dynamic test. A disadvantage might be, however, that information about the process of learning will be lost, for example, a previous study showed that increases in performance after training are most apparent when information had to be processed actively (Stevenson et al., 2016). Despite these considerations, the use of a computerized task may still be preferred, which is justified given the possibility to record information more accurately than with a paper-and-pencil test—it may be worth considering simplifying the buttons. In the current design, participants received a single explanation of the buttons at the beginning of each phase, and it is likely that they found it challenging to remember and internalize these instructions. Furthermore, the drag-and-drop system may have relied excessively on fine motor skills, suggesting that a more user-friendly interface could be sought in future implementations. Finally, in addition to including static neuropsychological tests, it would also be interesting to include information about personality characteristics and executive functioning in future research, such as motivation, cognitive flexibility, (test) anxiety, and/or conscientiousness, as previous studies have shown that these factors are associated with improvements on dynamic tests or learning in general (Huang & Bramble, 2016; Meijer, 2001; Stad et al., 2019; Tas et al., 2012).

## 5. Conclusions

We conclude that our results support the idea that static tests are not all-encompassing, underscoring the importance of exploring alternative means of administering neuropsychological tests. The dynamic test that was used in the current study is, however, in its currents form, suboptimal for use in this population. We found no group differences in this study, which may be due to the large variability in individual progression paths, supporting the idea that learning potential differs by individual. The exploratory initial results are promising and suggest that incorporating a dynamic test into standardized neuropsychological assessment could be of added value to measure learning potential. Therefore, research on dynamic testing in this heterogeneous clinical psychiatric population needs further investigation.

## Figures and Tables

**Table 1:** Study design.

Group	Static Test Measures			Dynamic Test Measures			
	WAIS-IV-NL	RAVLT	LLT	Pretest	Training	Practice	Posttest
Training group (*n* = 14)	X	X	X	X	X		X
Practice group (*n* = 15)	X	X	X	X		X	X
Control group (*n* = 11)	X	X	X	X			X

WAIS-IV-NL, fourth edition of the Dutch Version of the Wechsler Adult Intelligence Scale; RAVLT, Rey Auditory Verbal Learning Test; LLT, Location Learning Test.

**Table 2:** Used variables for the static measures.

Test	Variable M ± SD (min–max)	Meaning
WAIS-IV-NL	Full-Scale IQ 100 ± 15 (40–160)	Intelligence quotient, corrected for age and made up of the four index scores below
	Verbal Comprehension Index 100 ± 15 (40–160)	Index score, corrected for age
	Perceptual Comprehension Index 100 ± 15 (40–160)	Index score, corrected for age
	Working Memory Index 100 ± 15 (40–160)	Index score, corrected for age
	Processing Speed Index 100 ± 15 (40–160)	Index score, corrected for age
RAVLT	Total score (0–75)	The total number of correct answers on all five trials
LLT	Total score (0–305)	The total number of errors on all five trials
	Learning index (0–1)	The total of the improvement ratios between the trials one to five, divided by four

WAIS-IV-NL, fourth edition of the Dutch Version of the Wechsler Adult Intelligence Scale; RAVLT, Rey Auditory Verbal Learning Test; LLT, Location Learning Test.

**Table 3:** Descriptives of the administration times in minutes on the dynamic test.

Phase	*n*	Minimum	Maximum	Mean	Standard Deviation
Pretest	38	3.27	19.38	11.58	3.49
Training	14	4.80	29.81	14.52	6.32
Practice	15	3.90	10.91	6.96	2.12
Posttest	40	3.56	19.49	9.33	3.29

Time scores represent only the recorded attempts and exclude the time spent on responses that participants deleted.

**Table 4:** Descriptives of the dynamic and static measures.

Dynamic Measures	Minimum-Maximum	Training Group (*n* = 14, M ± SD (Range))	Practice Group (*n* = 15, M ± SD (Range))	Control Group (*n* = 11, M ± SD (Range))
Pretest total score	0–10	3.07 ± 3.22 (0–10)	3.40 ± 2.61 (0–8)	4.73 ± 2.61 (0–8)
Posttest total score	0–10	6.64 ± 3.50 (0–10)	6.00 ± 3.48 (0–10)	6.91 ± 2.59 (0–10)
**Static Measures**	**Minimum-Maximum**	**Training Group (*n* = 14, M ± SD (Range))**	**Practice Group (*n* = 15, M ± SD (Range))**	**Control Group (*n* = 11, M ± SD (Range))**
WAIS-IV-NL FSIQ	45–155	98.39 ± 17.20 (90–107) ^1^	91.00 ± 12.48 (83–99)	101.50 ± 14.53 (92–111) ^3^
WAIS-IV-NL VCI	45–155	103.46 ± 14.81 (66–120) ^1^	97.67 ± 8.80 (87–116)	102.50 ± 15.92 (68–118) ^3^
WAIS-IV-NL PRI	45–155	97.85 ± 18.92 (60–121) ^1^	97.73 ± 17.67 (72–127)	100.90 ± 15.11 (81–131) ^3^
WAIS-IV-NL WMI	45–155	101.62 ± 15.41 (61–117) ^1^	90.80 ± 11.19 (74–117)	100.50 ± 12.14 (86–117) ^3^
WAIS-IV-NL PSI	45–155	90.62 ± 21.26 (63–141) ^1^	87.40 ± 13.15 (70–122)	99.90 ± 14.08 (76–128) ^3^
RAVLT total score	0–75	45.31 ± 11.66 (20–62) ^1^	47.86 ± 8.92 (32–60) ^2^	54.50 ± 4.74 (47–63) ^3^
LLT total score	0–175	24.69 ± 20.25 (0–59) ^1^	13.73 ± 10.96 (1–41)	10.11 ± 9.97 (0–31) ^4^
LLT learning index	0–1	0.71 ± 0.24 (0.34–1) ^1^	0.70 ± 0.27 (0.18–1)	0.88 ± 0.23 (0.32–1) ^4^

^1^*n* = 13; ^2^ *n* = 14; ^3^ *n* = 10; ^4^ *n* = 9; note that some participants had incomplete data, which resulted from methodological constraints of clinical research and were unrelated to participant characteristics. WAIS-IV-NL, fourth edition of the Dutch Version of the Wechsler Adult Intelligence Scale; FSIQ, Full-Scale Intelligence Quotient; VCI, Verbal Comprehension Index; PRI, Perceptual Reasoning Index; WMI, Working Memory Index; PSI, Processing Speed Index; RAVLT, Rey Auditory Verbal Learning Test; LLT, Location Learning Test.

**Table 5:** Correlation matrix.

Test	Variable	All Conditions Pretest		Training Group Posttest		Practice Group Posttest		Control Group Posttest	
		*r*	*p*	*r*	*p*	*r*	*p*	r	*p*
Dynamic test	Pretest			0.610	0.021	0.879	<0.001	0.795	0.003
	Posttest	0.730	<0.001						
WAIS-IV-NL	FSIQ	0.715	<0.001	0.572	0.041	0.624	0.013	0.861	0.001
	VCI	0.557	<0.001	0.533	0.061	0.545	0.036	0.790	0.646
	PRI	0.675	<0.001	0.568	0.043	0.661	0.007	0.699	0.024
	WMI	0.565	<0.001	0.613	0.026	0.482	0.069	0.878	0.001
	PSI	0.507	0.001	0.252	0.407	0.390	0.151	0.415	0.234
RAVLT	Total score	0.510	0.001	0.479	0.098	0.510	0.062	0.252	0.483
LLT	Total score	−0.407	0.012	−0.135	0.661	−0.770	0.001	−0.524	0.148
	Learning index	0.525	0.001	0.257	0.397	0.710	0.003	0.629	0.070

WAIS-IV-NL, fourth edition of the Dutch Version of the Wechsler Adult Intelligence Scale; FSIQ, Full-Scale IQ; VCI, Verbal Comprehension Index; PRI, Perceptual Reasoning Index; WMI, Working Memory Index; PSI, Processing Speed Index; RAVLT, Rey Auditory Verbal Learning Test; LLT, Location Learning Test.

## Data Availability

Anonymized data presented in this study are available on request from the corresponding author, since the data are not publicly available due to privacy restrictions.

## Abbreviations

The following abbreviations are used in this manuscript:

FSIQ	Full-Scale Intelligence Quotient
LLT	Location Learning Test
MCI	Mild Cognitive Impairment
PRI	Perceptual Reasoning Index
PSI	Processing Speed Index
RAVLT	Rey Auditory Verbal Learning Test
VCI	Verbal Comprehension Index
WAIS-IV-NL	Fourth edition of the Dutch version of the Wechsler Adult Intelligence Scale
WMI	Working Memory Index
ZPD	Zone of Proximal Development
